# Postoperative Clinical Outcomes of Transurethral Resection of the Prostate (TURP) Combined With Otis Urethrotomy in High-Risk Patients With Coronary Artery Disease (Post-Angioplasty): Insights From a Prospective Observational Cohort

**DOI:** 10.7759/cureus.91439

**Published:** 2025-09-01

**Authors:** A Yagneshwar Sharma, Bhrigudutt Tiwari, Shubha Ekka, Abhishek Kumar, Murlidhar Jangir, Mamta Peswani, Siddharth Agrawal

**Affiliations:** 1 Department of Urology, Chhattisgarh Institute of Medical Sciences, Bilaspur, IND; 2 Department of General Surgery, Chhattisgarh Institute of Medical Sciences, Bilaspur, IND; 3 Department of Cardiology, Chhattisgarh Institute of Medical Sciences, Bilaspur, IND

**Keywords:** benign prostatic hyperplasia, coronary artery disease, dual antiplatelet therapy, otis urethrotomy, transurethral resection of the prostate, urethral stricture

## Abstract

Background

Transurethral resection of the prostate (TURP) is widely used for treating benign prostatic hyperplasia (BPH) but is associated with postoperative urethral strictures, especially in patients with narrow urethral anatomy. The risk is further complicated in patients with coronary artery disease (CAD) on dual antiplatelet therapy, where surgical bleeding and cardiac events are significant concerns. Otis urethrotomy may help reduce stricture risk, but its safety and effectiveness in high-risk cardiac patients undergoing TURP remain underexplored.

Materials and methods

In this prospective observational study, 80 male CAD patients with prior angioplasty underwent TURP with prophylactic Otis urethrotomy. All patients were assessed and optimized preoperatively through a multidisciplinary approach. Follow-up evaluations at one, three, and six months included assessments of urinary flow (Qmax), post-void residual (PVR) urine, International Prostatic Symptom Score (IPSS), quality of life (QOL) score, and perioperative complications.

Results

Urethral stricture occurred in only two patients (2.5%), notably lower than typical post-TURP rates. Qmax improved from 7.1 to 12.3 mL/s, and PVR declined from 165.5 to 52.7 mL. IPSS decreased from 23.5 to 14.3, and QOL scores improved from 4.5 to 2.0. Cardiac complications were limited to minor events (angina 2.5%, arrhythmia 1.2%), with no reinfarctions. Urological complications were mild and self-limiting.

Conclusion

The combination of Otis urethrotomy with TURP in CAD patients is safe and effective, yielding strong functional outcomes and low complication rates. It offers a promising surgical option in high-risk individuals when managed through a structured, multidisciplinary framework.

## Introduction

Transurethral resection of the prostate (TURP) remains one of the most commonly performed surgical procedures in urology, widely accepted for relieving bladder outlet obstruction caused by benign prostatic hyperplasia (BPH). The standard instrument used for this procedure is a 24 or 26 French (Fr) resectoscope. However, in the Indian population, the urethra is often anatomically narrower than in Western men, which frequently demands dilation of the urethral meatus and navicular fossa to accommodate the instrument [1]. The outer sheath of the resectoscope features fenestrations for continuous irrigation, but repeated in-and-out movement during the procedure can lead to mucosal trauma, often referred to as the cheese grater effect [2]. This effect occurs when the urethral mucosa is pulled into the fenestrations and damaged by friction. Such trauma, particularly when combined with the ischemic insult caused by large and rigid instruments, contributes significantly to the development of urethral strictures. These strictures commonly occur at the meatus, penoscrotal junction, and bulbar urethra-regions most vulnerable to mechanical injury and reduced blood supply [3]. Despite advancements in surgical techniques and instrumentation, postoperative urethral strictures continue to affect approximately 6%-8% of patients, leading to urinary obstruction, discomfort, and a considerable impact on quality of life (QOL) [3]. Over the years, various methods have been explored to reduce the risk of stricture formation following TURP. One such intervention is an Otis urethrotomy, a mechanical dilation technique designed to expand the anterior urethra, allowing for a smoother passage of the resectoscope. Although earlier studies have reported mixed results regarding its effectiveness, some have demonstrated a modest reduction in urethral stricture incidence when Otis urethrotomy was performed before TURP [4]. However, consistent evidence is lacking, and most studies have failed to evaluate the safety of this practice in specific high-risk patient groups.

A distinct feature of the present study is its exclusive focus on a high-risk population-patients with coronary artery disease (CAD) who have previously undergone percutaneous coronary intervention (PCI), more commonly known as coronary angioplasty. Managing urological surgical procedures such as TURP in patients with established CAD poses significant clinical challenges. These individuals are widely prescribed dual antiplatelet therapy (DAPT)-typically aspirin combined with a P2Y12 inhibitor like clopidogrel or ticagrelor-to prevent stent thrombosis following angioplasty. While this therapy is essential for maintaining cardiac stent patency and preventing major adverse cardiovascular events, it significantly complicates the perioperative period. Continuation of antiplatelet therapy increases the risk of excessive intraoperative and postoperative bleeding, especially with procedures involving highly vascular areas such as the prostate. On the contrary, temporary discontinuation of antiplatelet drugs-particularly within the first three to 12 months after PCI-substantially elevates the risk of acute stent thrombosis, myocardial infarction, and even sudden cardiac death [5]. This presents a complex dilemma for both urologists and cardiologists, who must weigh the relative risks of bleeding against those of thrombotic events. In such settings, the addition of another instrumentation procedure, such as Otis urethrotomy, may further raise concerns regarding potential bleeding and perioperative hemodynamic instability. Moreover, CAD patients often have reduced cardiac reserve and poor tolerance to stress, both surgical and anesthetic. Hemodynamic fluctuations during spinal anesthesia or blood loss during TURP can precipitate ischemia, arrhythmias, or heart failure. Despite these complexities, CAD patients represent a growing subset of surgical candidates for TURP due to increasing life expectancy and improved cardiovascular interventions. However, there is a lack of focused research addressing the safety and efficacy of adjunctive procedures like Otis urethrotomy in this subgroup. Most previous studies on prophylactic urethrotomy did not control for cardiac comorbidities, and the real-world risk-benefit ratio in CAD patients remains undefined. This study, therefore, aims to evaluate both the efficacy of Otis urethrotomy in preventing postoperative urethral strictures and the safety of performing this intervention in a cardiac-compromised population on antiplatelet therapy. By systematically assessing both urological and cardiovascular outcomes, this research was intended to fill an important gap in the literature and support safer and more individualized perioperative planning for TURP in patients with significant cardiac risk.

## Materials and methods

Study design

This was a prospective observational study conducted at Chhattisgarh Institute of Medical Sciences, Bilaspur, a tertiary care center located in the central part of India, serving a predominantly tribal population from December 2020 to October 2024. The study period was extended due to the COVID-19 pandemic.

Objectives

The primary objective of the study was to evaluate how effective Otis urethrotomy is in preventing urethral stricture after TURP. The secondary objectives were to monitor for complications related to the use of Otis urethrotomy in CAD patients, which include (1) urological complications, such as bleeding, urinary incontinence, erectile dysfunction, and penile swelling, and (2) cardiovascular complications, such as myocardial infarction, arrhythmia, and heart failure occurring during and after surgery.

Inclusion criteria

The study included male patients who had been diagnosed with CAD and had previously undergone PCI, commonly referred to as angioplasty. All participants were scheduled to undergo TURP for the management of symptomatic BPH. Before inclusion, each patient was thoroughly assessed and deemed fit for surgery under spinal anesthesia following clearance from a cardiologist, ensuring optimal perioperative safety for this cardiac-compromised group. Furthermore, all individuals provided written informed consent and agreed to comply with the follow-up schedule, demonstrating their willingness to participate fully in the study protocol.

Exclusion criteria

Patients were excluded based on the following criteria: (1) prior history of urethral manipulation or instrumentation; (2) documented previous urethral stricture; (3) meatal stenosis; (4) history of urethritis (infectious or non-infectious); (5) history of urethral stone passage; (6) requirement of additional procedures during TURP, such as cystolithotripsy or litholapaxy; (7) histologically or radiologically proven prostate cancer; (8) prostate size greater than 100 g (determined by preoperative ultrasound) based on recent recommendations by Young et al., as larger prostates carry a higher bleeding risk and may require multiple surgical sessions, which was challenging in this CAD patient cohort [6]; (9) established urinary incontinence; and (10) prior history of erectile dysfunction.

Ethical and regulatory approvals

This study was conducted after obtaining approval from the Institutional Thesis Review Committee and the Institutional Ethics Committee. Before inclusion, all patients were informed in detail about the nature of the study, its objectives, potential benefits, and possible risks, following which written informed consent was obtained from each participant. The study adhered to the ethical principles outlined in the Declaration of Helsinki (2013 revision), ensuring respect for patient rights, safety, and well-being throughout the research process [7].

Preoperative evaluation and preparation

All enrolled patients underwent a thorough clinical history, symptom assessment, and physical examination and were documented by the treating urology surgeon at the time of admission. A multidisciplinary approach was employed for perioperative management, involving close coordination between the urology and cardiology teams to ensure patient safety. Baseline investigations included complete blood count, renal function tests, liver function tests, and coagulation profile. Prostate-specific antigen (PSA) levels were measured, and urinalysis, along with urine culture and sensitivity testing, was performed. Abdominal ultrasonography was used to assess prostate volume and post-void residual (PVR) urine, while a plain X-ray of the kidneys, ureters, and bladder (KUB) helped rule out urolithiasis. Cardiac investigation included electrocardiogram (ECG), echocardiogram, and high-sensitivity cardiac troponin I (hs-cTnI). Patients who were taking DAPT were managed as per the 2017 European Society of Cardiology (ESC) guidelines on DAPT for CAD for non-cardiac surgery [8]. If a patient had undergone angioplasty less than one month before, the TURP surgery was postponed until at least one month had passed. Since TURP is a low-risk surgery, all patients continued to take aspirin during the perioperative period, regardless of the type of stent (bare-metal or drug-eluting) or how long ago the angioplasty was done (as long as it was more than one month ago). Clopidogrel or other P2Y12 inhibitors were reconsidered on an individual basis. They were continued if the angioplasty had been done within the last year and the patient had a high risk of cardiac events. High-risk features included a history of stent thrombosis despite adequate antiplatelet therapy, stenting of the last remaining patent coronary artery, having diffuse disease in multiple vessels (especially in diabetic patients), chronic kidney disease (with creatinine clearance below 60 mL/min), placement of three or more stents, treatment of three or more lesions, stenting of bifurcations using two stents, total stent length over 60 mm, or treatment of a chronic total occlusion [8]. For patients who had their angioplasty more than one year ago, clopidogrel was usually stopped five days before surgery if they were on DAPT, but continued if they were on single antiplatelet therapy (SAPT) (either aspirin or clopidogrel alone). The Revised Cardiac Risk Index (RCRI) and HAS-BLED score were used in this study to assess perioperative risk. The RCRI evaluates six factors-history of ischemic heart disease, congestive heart failure, cerebrovascular disease, insulin-dependent diabetes mellitus, chronic kidney disease (creatinine > 2 mg/dL), and high-risk surgery-to estimate the risk of cardiac complications during non-cardiac surgery [9]. The HAS-BLED score assesses bleeding risk in patients, considering seven factors: hypertension, abnormal liver/renal function, stroke history, bleeding history or predisposition, labile international normalized ratio (INR), age over 65, and concomitant use of drugs or alcohol [9]. Each score helps stratify patients' risk and guide clinical management accordingly.

Surgical technique

All TURP procedures were performed by the same urology surgeon. They were carried out under regional (spinal) anesthesia using a standard 24 or 26 Fr continuous-flow resectoscope, with 1.5% glycine employed as the irrigation fluid. Before initiating the prostatic resection, a prophylactic Otis urethrotomy of 2-3 cm was performed in all patients using an Otis urethrotome set to 30 Fr, targeting the 12 o’clock position [10]. This step was undertaken to pre-emptively dilate the anterior urethra and thereby reduce the risk of ischemic injury caused by instrumentation during the procedure. After completion of the resection, a 22 Fr three-way Foley catheter was inserted in each patient to facilitate effective postoperative irrigation and bladder decompression [10]. Intraoperative parameters such as findings on cystourethroscopy-including any urethral anomalies or notable prostate morphology-were carefully documented, along with total resection time in minutes and any intraoperative complications encountered during the procedure. TURP blood loss was calculated by comparing the hemoglobin (Hb) concentration in the irrigation fluid to the patient's preoperative Hb and the total volume of irrigation fluid. A common formula is [10]

$\text{Estimated blood loss (mL)} =\frac{\text{Irrigation fluid volume (mL)} \times \text{Fluid Hb (g/dL)}}{\text{Patient's preoperative Hb (g/dL)}}$

Postoperative care and monitoring

Patients were monitored closely during the postoperative period, with particular attention given to both urological and cardiac complications. Key parameters observed included the ECG changes, high-sensitivity troponin values, duration of catheterization, time to ambulation, and the length of hospital stay until discharge. Any postoperative events such as bleeding, penile edema, urinary incontinence, erectile dysfunction, or the need for blood transfusion were carefully documented. In addition, cardiovascular events such as reinfarction, angina, or arrhythmias were actively monitored, given the cardiac history of all patients. Angina is typically identified by chest pain or discomfort that may radiate to the neck, jaw, shoulder, or arms-especially during exertion-and is confirmed with an ECG or blood tests for cardiac markers. Arrhythmias are suspected based on symptoms like palpitations, dizziness, or syncope and diagnosed by ECG or sometimes Holter monitoring. Common examples of arrhythmias include atrial fibrillation, ventricular tachycardia, supraventricular tachycardia, sinus bradycardia, and premature ventricular contractions. Special focus by the treating urology surgeon was placed on identifying early signs of urethral stricture formation, including symptoms like weakened urinary stream, urinary hesitancy, and a sensation of incomplete bladder emptying.

Follow-up protocol

Patients were systematically followed up at one, three, and six months after surgery to monitor their recovery and detect any postoperative complications. At each scheduled visit, a comprehensive urological evaluation was conducted by a fellow urology surgeon other than the one who had performed the surgery, and hence, blinding was applied. The International Prostatic Symptom Score (IPSS) was used to assess the severity of lower urinary tract symptoms in BPH patients [11]. It includes seven symptom-related questions and one QOL question, with scores ranging from 0 to 35. Scores of 0-7 indicate mild, 8-19 moderate, and 20-35 severe symptoms, allowing objective evaluation before and after surgery. The QOL score-derived from a single IPSS question-was used to assess the impact of urinary symptoms on patients' daily life [12]. It ranges from 0 (delighted) to 6 (terrible), with lower scores indicating better perceived QOL. This helped evaluate symptom relief and patient satisfaction after surgery. Uroflowmetry was performed at every visit, with particular focus on measuring the maximum urinary flow rate (Qmax) and assessing PVR urine volume. The development of a urethral stricture was suspected if patients exhibited a reduction in Qmax to less than 10 mL/s, accompanied by obstructive urinary symptoms. The suspected cases of stricture during follow-up were confirmed by X-ray retrograde urethrogram.

Statistical analysis

Data collected during the study were entered into Microsoft Excel™ (Microsoft Corp., Redmond, WA, US) and analyzed using SPSS version 27.0 (IBM Corp., Armonk, NY, US). Descriptive statistical methods were employed to summarize the findings. Continuous variables were expressed as mean ± standard deviation (SD), while categorical variables were presented as frequencies and percentages. The sample size was estimated using the standard formula: n = t^2^ × p(1 − p)/m^2^, where n represents the required sample size, t is the standard normal deviate at 95% confidence level (1.96), p is the expected prevalence, and m is the margin of error (0.05). Based on these parameters and calculations from available data, a sample size of 80 patients was determined to be adequate to achieve the study’s objectives with sufficient statistical power.

## Results

The study cohort had a mean age of 66.8 years, with most patients (56.2%) on SAPT and 43.8% on DAPT. Common comorbidities included hypertension (63.7%), diabetes (40.0%), dyslipidemia (47.5%), smoking history (35.0%), and alcohol use (30.0%). A significant proportion had complex coronary disease, with 66.3% having two or more stents. The average time since PCI was 13.6 months. The mean preoperative hs-cTnI level was 9.2 ng/L, suggesting no active myocardial injury. The average prostate volume was 58.2 g, and the PSA level was within the benign range (2.9 ng/mL). Urodynamic parameters reflected severe lower urinary tract obstruction, with a reduced Qmax of 7.1 mL/s, high PVR (165.5 mL), elevated IPSS (23.5), and poor QOL score (4.5) (Table 1). Overall, this baseline profile indicates an elderly, high-risk population with significant cardiac and urological disease burden. The mean RCRI score is 2.8 ± 1.1, with a range from 1 to 5, and the mean HAS-BLED score is 3.6 ± 1.2, ranging from 2 to 6. All patients were on aspirin, and 14 (17.5%) patients were on DAPT (aspirin and clopidogrel) at the time of surgery.

**Table 1:** Baseline demographic, clinical, and laboratory profile of the study cohort, showing distribution and variability PCI: percutaneous coronary intervention; SAPT: single antiplatelet therapy; DAPT: dual antiplatelet therapy; PSA: prostate-specific antigen; Qmax: maximum urinary flow rate; PVR: post-void residual urine; SD: standard deviation

Parameter	Mean ± SD/frequency (%)	Minimum	Maximum
Age (years)	66.8 ± 5.7	49.7	83.9
History of PCI (months)	13.6 ± 4.2	1.0	26.2
Antiplatelet regimen: SAPT	45 (56.2%)	–	–
Antiplatelet regimen: DAPT	35 (43.8%)	–	–
Diabetes mellitus	32 (40.0%)	–	–
Hypertension	51 (63.7%)	–	–
Smoking history	28 (35.0%)	–	–
Alcohol consumption	24 (30.0%)	–	–
Dyslipidemia	38 (47.5%)	–	–
Stent distribution: single stent	27 (33.7%)	–	–
Stent distribution: double stent	25 (31.2%)	–	–
Stent distribution: triple stent	15 (18.8%)	–	–
Stent distribution: >three stents	13 (16.2%)	–	–
High-sensitivity cardiac troponin I (hs-cTnI) (ng/L)	9.2 ± 4.5	0.1	22.7
PSA level (ng/mL)	2.9 ± 1.4	0.1	7.1
Prostate volume (g)	58.2 ± 7.5	35.7	80.7
Preoperative Qmax (mL/s)	7.1 ± 1.9	1.4	12.8
Preoperative PVR (mL)	165.5 ± 24.8	91.1	239.9
International Prostatic Symptom Score (IPSS)	23.5 ± 3.4	13.3	33.7
Quality of life (QOL) score	4.5 ± 0.6	2.7	6.3
Revised Cardiac Risk Index (RCRI)	2.8 ± 1.1	1	5
HAS-BLED score	3.6 ± 1.2	2	6

The mean operative time was 41.2 ± 9.3 minutes, and the average blood loss was 68.5 ± 15.4 mL. Catheter duration averaged 7.2 ± 2.3 days. All patients received a standardized 22 Fr three-way Foley catheter. Postoperative hs-cTnI levels had a mean value of 17.5 ± 8.5 ng/L, with a range from 0.1 to 38.0 ng/L (Table 2).

**Table 2:** Intraoperative and postoperative parameters with distribution and variability (n = 80) SD: standard deviation; hs-cTnI: high-sensitivity cardiac troponin I

Parameter	Mean ± SD/frequency (%)	Minimum	Maximum
Operative time (minutes)	41.2 ± 9.3	13.3	69.1
Blood loss (mL)	68.5 ± 15.4	22.3	114.7
Catheter duration (days)	7.2 ± 2.3	3.5	15.0
Foley catheter size	22 Fr (3-way)	22 Fr	22 Fr
Postoperative hs-cTnI (ng/L)	17.5 ± 8.5	0.1	38.0

**Figure 1:**
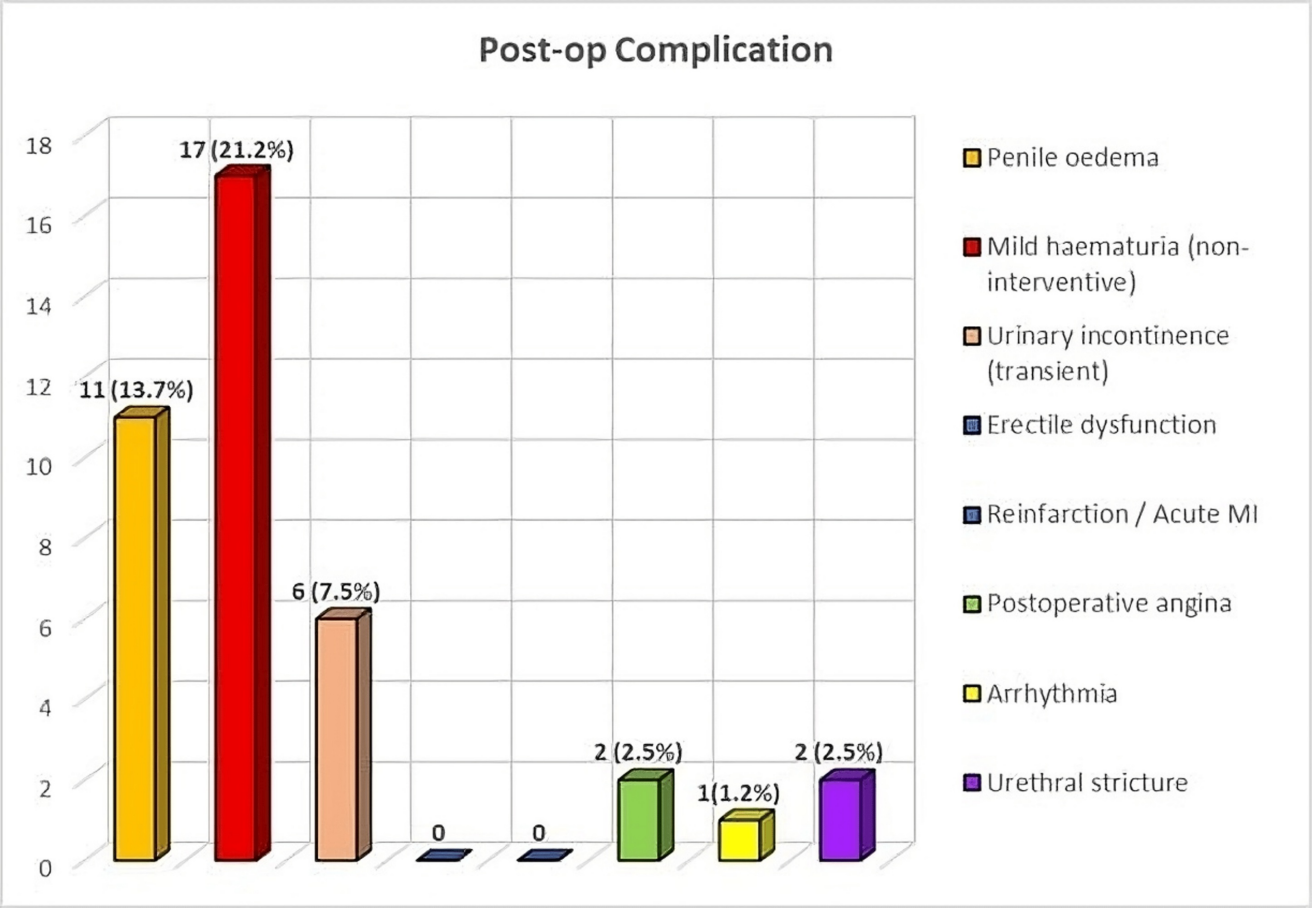
The bar chart displaying the frequency of various postoperative complications (n (%)) MI: myocardial infarction

Mild, self-limiting complications were the most frequently observed, with penile edema and mild hematuria occurring in 13.7% and 21.2% of cases, respectively. Transient urinary incontinence was noted in a small proportion (7.5%) and resolved over time. Importantly, there were no instances of erectile dysfunction or reinfarction/acute myocardial infarction. Cardiac events such as postoperative angina and arrhythmia were rare (2.5% and 1.2%, respectively), indicating a low incidence of serious adverse events in this high-risk population (Table 3, Figure 1).

**Table 3:** Postoperative complications MI: myocardial infarction

Complication	Frequency (%)
Penile edema	11 (13.7%)
Mild hematuria (non-interventive)	17 (21.2%)
Urinary incontinence (transient)	6 (7.5%)
Erectile dysfunction	0 (0%)
Reinfarction/acute MI	0 (0%)
Postoperative angina	2 (2.5%)
Arrhythmia	1 (1.2%)
Urethral stricture	2 (2.5%)

**Figure 2:**
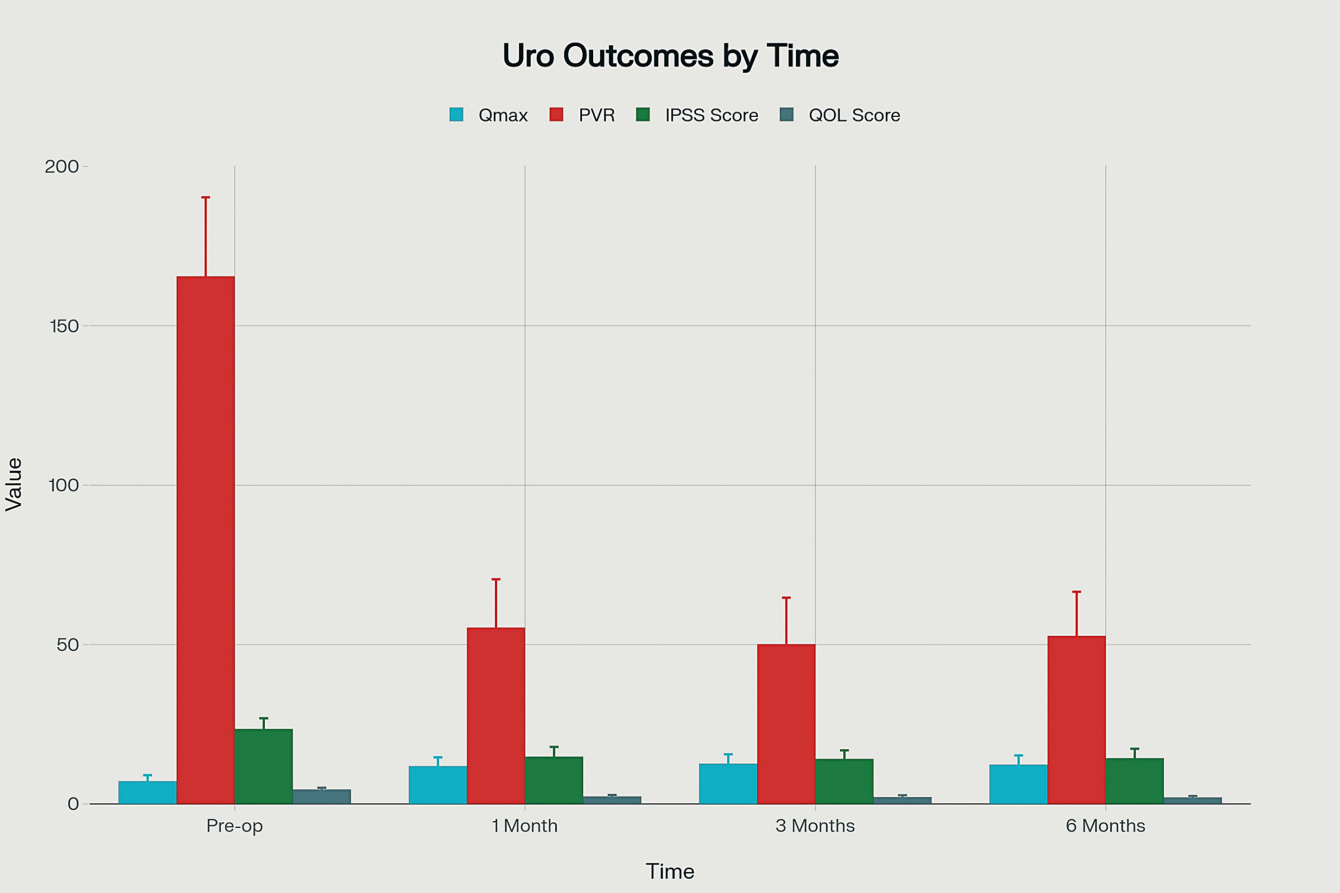
Bar chart illustrating the mean and standard deviation of four urological outcomes (Qmax, PVR, IPSS, and QOL score) at pre-op, 1 month, 3 months, and 6 months Qmax: maximum urinary flow rate; PVR: post-void residual urine volume; IPSS: International Prostate Symptom Score; QOL: quality of life score

In this study of 80 patients undergoing TURP, only two patients (2.5%) developed postoperative urethral stricture during follow-up. The analysis of functional urological outcomes over time demonstrated significant and sustained improvements across all measured parameters. The maximum Qmax increased from a preoperative mean of 7.1 ± 1.9 mL/s to 12.3 ± 2.9 mL/s at six months (p = 0.0012), indicating enhanced voiding efficiency. PVR showed a marked reduction from 165.5 ± 24.8 mL preoperatively to 52.7 ± 13.9 mL at six months (p = 0.0005). Symptom severity, assessed using the IPSS, improved significantly from 23.5 ± 3.4 to 14.3 ± 3.0 (p = 0.0031), reflecting a shift from severe to moderate symptom range. Similarly, patients’ QOL scores improved from 4.5 ± 0.6 to 2.0 ± 0.5 (p = 0.0014), indicating better satisfaction and reduced symptom-related discomfort (Figure 2, Table 4).

**Table 4:** Functional urological outcomes over time with ANOVA summary Qmax: maximum urinary flow rate; PVR: post-void residual urine volume; IPSS: International Prostate Symptom Score; QOL: quality of life score

Outcome	Pre-op	1 month	3 months	6 months	F-value	p-value
Qmax (mL/s)	7.1 ± 1.9	11.8 ± 2.8	12.6 ± 3.0	12.3 ± 2.9	5.85	0.0012
PVR (mL)	165.5 ± 24.8	55.3 ± 15.2	50.1 ± 14.6	52.7 ± 13.9	7.23	0.0005
IPSS	23.5 ± 3.4	14.8 ± 3.1	14.1 ± 2.7	14.3 ± 3.0	4.97	0.0031
QOL score	4.5 ± 0.6	2.3 ± 0.5	2.1 ± 0.6	2.0 ± 0.5	6.12	0.0014

## Discussion

Patient demographics, baseline characteristics, and cardiovascular profile

The mean age of the cohort was 66.8 ± 5.7 years, consistent with the documented epidemiology of BPH and CAD, both of which are strongly age-associated pathologies. In a multinational analysis by Wang et al., the prevalence of moderate-to-severe lower urinary tract symptoms increased sharply after the sixth decade in men, mirroring our population demographics [13]. Moreover, the average time since PCI was 13.6 ± 4.2 months. This means most patients were beyond the period when stent thrombosis risk is highest. However, they still needed regular monitoring and medication. This is shown by the fact that 56.2% were taking a SAPT, while 43.8% were continued on DAPT. The presence of other major cardiovascular risk factors-diabetes mellitus (40.0%), hypertension (63.7%), and dyslipidemia (47.5%)-underlines the multifactorial cardiovascular burden of our cohort. These rates surpass those reported in the general urological surgical population and rival findings from large cardiovascular outcome trials such as the REACH Registry [14]. Notably, a significant portion of the patients were active or former smokers (35.0%), and alcohol consumption was reported in 30.0%-factors known to delay tissue healing and increase perioperative morbidity. While our study design excluded patients with known urethral comorbidities, other metabolic comorbidities can still indirectly impair urethral vascularity and healing. An important observation from our data is the diversified stent profile: 33.7% had a single stent, but a considerable proportion had multiple (≥2) stents (66.3%). This distribution underscores a high burden of coronary disease and, importantly, a proportionally higher dependence on DAPT even after one year post-PCI [9]. Interestingly, hs-cTnI measured preoperatively was within subcritical ranges (mean 9.2 ± 4.5 ng/L), suggesting no active ischemia in most cases. According to recent cardiology literature, elevations < 19 ng/L, especially without clinical symptoms or ECG changes, are unlikely to represent ongoing myocardial injury [15]. The mean RCRI score of 2.8 ± 1.1, with a range from 1 to 5, indicates a predominantly moderate to high perioperative cardiac risk in our cohort of patients. The inclusion of this marker further supports the judicious patient selection and optimization prior to urological surgery.

Preoperative urological parameters: baseline disease severity and intervention rationale

Preoperative PSA levels averaged 2.9 ± 1.4 ng/mL, which falls within the expected range in patients with BPH but without overt malignancy, aligning with the European Association of Urology (EAU) Guidelines that suggest PSA levels in the 1.5-4.0 ng/mL range are common in uncomplicated BPH [14]. The mean prostate volume was 58.2 ± 7.5 g, supporting TURP as an appropriate intervention, especially as volumes exceeding 80 g are better addressed via open prostatectomy or laser vaporization techniques per EAU guidelines [16]. The clinical and urodynamic findings provide a clear rationale for operative management. The mean Qmax of 7.1 ± 1.9 mL/s is significantly below normal cutoffs (typically >15 mL/s), denoting severe obstruction and reduced detrusor contractility. The mean PVR of 165.5 ± 24.8 mL signifies significant post-void urine retention, raising concerns regarding the risk of upper tract damage, recurrent urinary tract infections, and progressive bladder dysfunction if left untreated. A high mean IPSS of 23.5 ± 3.4, reflecting severe lower urinary tract symptoms, coupled with a QOL score of 4.5 ± 0.6, highlights substantial symptom burden and impaired daily functioning. These measures, which have been validated as predictors of poor QOL and indicators for surgery, are consistent with findings from Garg et al., who emphasized that low Qmax and high PVR/IPSS strongly predict the need for surgical intervention [17]. Few studies have examined the efficacy of Otis urethrotomy in preventing post-TURP strictures, particularly in high-risk or instrumentally challenged populations. Selius and Subedi reported a stricture rate reduction from 9.1% to 4.3% when Otis urethrotomy was used as a preventive step during TURP in elderly men [18]. However, these studies did not account for cardiac comorbidities or perioperative antithrombotic therapies.

Intraoperative and immediate postoperative outcomes: safety and efficiency considerations

The mean operative time (41.2 ± 9.3 minutes) is notable for its efficiency, falling well within international averages for TURP in moderate-sized prostates and highlighting the proficiency of the operative urology surgeon [19]. Operative duration is particularly relevant in cardiac patients, as shorter procedures are associated with lower anesthesia-related risks and decreased chances of perioperative cardiac events. Blood loss, averaging 68.5 ± 15.4 mL, is very modest, and intraoperative safety was good, especially considering that all patients were on SAPT and a few patients (17.5%) were on DAPT during the operation. Previous studies, such as that by Rassweiler et al., have reported much higher average blood loss in standard TURP, especially in non-anticoagulated Western cohorts [20]. The study's low blood loss suggests that with careful technique, including the use of a single dorsal Otis urethrotomy (minimizing vascular injury), bleeding can be kept within safe limits even in high-risk settings. Otis urethrotomy was performed at the 12 o’clock position to minimize vascular trauma-a technique endorsed by previous studies for safer outcomes [2]. Moreover, the avoidance of deeper lateral incisions likely contributed to a lower complication rate.

Postoperative complications: risk assessment and explanatory reasoning

The observed postoperative complication rates were low and non-severe: mild hematuria in 21.2%, penile edema in 13.7%, and transient urinary incontinence in 7.5%, all consistent with or better than previously published series of standard TURP. For example, Chen et al. described incontinence rates between 5% and 8% and higher post-TURP bleeding, supporting the idea that Otis urethrotomy did not add significant urological risk [21]. The absence of erectile dysfunction is also significant, as Otis urethrotomy was strictly midline (12 o’clock), thereby avoiding lateral urethral neurovascular bundles as validated by Urkmez et al. [22]. This surgical distinction is an important safeguard for functional preservation. Catheter duration averaged 7.2 ± 2.3 days, matching recommendations from evidence-based reviews that suggest 5-7 days is optimal for promoting mucosal healing, limiting stricture development, and reducing infection rates [6]. However, few patients do require a longer duration of catheterization due to minor bleeding caused by the continuation of antiplatelet therapy. The usage of a standardized 22 Fr three-way catheter ensures effective continuous irrigation and bladder decompression while minimizing additional trauma. In this study, postoperative cardiac complications were minimal despite the high-risk nature of the cohort, reflecting the effectiveness of careful perioperative management. Among 80 patients with significant CAD, only two cases (2.5%) of postoperative angina and one instance (1.2%) of arrhythmia (atrial fibrillation) were observed, with no incidents of reinfarction or perioperative myocardial infarction. This low complication rate is particularly noteworthy given the continued use of antiplatelet agents, which are essential for preventing stent thrombosis but increase surgical bleeding risk. Additionally, hs-cTnI measurements offered valuable insight into myocardial stress response. While postoperative levels increased modestly (mean 17.5 ± 8.5 ng/L from a preoperative mean of 9.2 ± 4.5 ng/L), they remained well below the threshold for myocardial infarction (≥52 ng/L), and none of the elevations corresponded with clinical or ECG evidence of ischemia [23]. The absence of major cardiac events (no reinfarction, rare angina, and infrequent arrhythmia) provides strong reassurance regarding the cardiac safety of this protocol. It also illustrates the importance of perioperative collaboration between urologists and cardiologists in patient selection, medication management, anesthesia planning, and post-procedure monitoring. These findings counter concerns raised in some literature combining Otis urethrotomy with TURP, particularly in anticoagulated or anatomically narrow patients. Instead, as discussed by Abdeen et al., such an approach, especially when incisions are limited in depth and location, actually reduces friction-induced mucosal injury and may decrease the risk of late strictures [24].

Functional outcomes: clinical relevance and future directions

This study demonstrated marked improvements in all major urological outcomes after TURP combined with prophylactic Otis urethrotomy, supported by consistent numerical data throughout the six-month follow-up. At baseline, the average Qmax was 7.1 ± 1.9 mL/s, reflecting severe obstruction. By six months postoperatively, Qmax increased to 12.3 ± 2.9 mL/s-representing a 73% improvement-which correlates strongly with easier voiding and symptomatic relief. PVR, an indicator of bladder emptying efficiency, fell sharply from 165.5 ± 24.8 mL preoperatively to 52.7 ± 13.9 mL at follow-up, showing restored and sustained bladder function. Symptom burden, measured by the IPSS, dropped from a preoperative average of 23.5 ± 3.4 to 14.3 ± 3.0 at six months, transitioning patients from a severe to a mild symptom category and showing meaningful improvement. QOL scores also reflected this trend, moving from 4.5 ± 0.6 before surgery to 2.0 ± 0.5 postoperatively, which indicates substantial relief from urinary symptoms impacting daily activities. These results are consistent with findings by Deininger et al., who reported that symptomatic and flow rate improvements after TURP are generally most pronounced within the first three months and stable thereafter [25]. Two patients (2.5%) developed postoperative urethral stricture during follow-up. This stricture rate is notably lower than figures commonly reported for standard TURP without Otis urethrotomy, where studies have documented rates ranging from 4% to 8% in high-risk groups. For example, Schultz et al. in a controlled randomized trial found a 4% stricture rate after Otis internal urethrotomy compared to 16% with catheter dilation, highlighting the potential benefit of Otis urethrotomy in reducing strictures after prostate surgery [26]. Similarly, Komura et al. observed a stricture rate of 6.6% without Otis urethrotomy in diabetic patients [27]. These results suggest that the use of Otis urethrotomy can contribute to the lower incidence of strictures observed in high-risk CAD patients on continued antiplatelet therapy, without increasing major complication rates. Rehabilitation after TURP involves a gradual return to activity over 4-6 weeks, focusing on light exercise like walking and pelvic floor (Kegel) exercises to improve bladder control [28,29]. Enhanced recovery after surgery (ERAS) is an evidence-based, multidisciplinary care pathway aimed at reducing the impact of surgical stress, thereby accelerating postoperative recovery and finally reducing postoperative distress [29].

Limitations

This study provides valuable insights but has several key limitations. Being a single-center study limits its generalizability, and the absence of a control group makes it difficult to attribute benefits solely to the Otis urethrotomy. The six-month follow-up may not capture late-onset complications, and the small sample size limits the detection of rare events. Excluding patients with complex urological conditions narrows applicability to the broader TURP population. Reliance on subjective scores like IPSS and QOL introduces potential bias, and long-term cardiac outcomes were not assessed. These factors highlight the need for larger, multicenter trials with longer follow-up.

## Conclusions

This study shows that adding prophylactic Otis urethrotomy before TURP lowers postoperative urethral stricture rates to 2.5% in CAD patients on antiplatelet therapy, better than rates in similar high-risk groups. Patients had significant improvements in urinary flow, symptoms, and QOL at six months with only minor, manageable complications and no longer surgery or recovery time. It is, hence, recommended to use Otis urethrotomy, especially in patients with narrow anterior urethras, to reduce strictures without increasing bleeding. Careful patient selection-excluding those with larger prostate size or prior urethral issues-is important. Surgical technique should limit urethrotomy depth and avoid lateral injury to preserve function. Multidisciplinary collaboration between urology and cardiology, and preoperative risk assessment with RCRI and HAS-BLED scores, helps optimize care. Aspirin is generally continued perioperatively, while P2Y12 inhibitors are managed case-by-case. Perioperative cardiac monitoring with markers and ECG is recommended to promptly address complications.
